# Fanconi syndrome developing and worsening during anti-myeloma therapy in multiple myeloma: a case report and literature review

**DOI:** 10.3389/fonc.2026.1735528

**Published:** 2026-03-11

**Authors:** Pengpeng Zhang, Peng Li, Xinlian Zhang, Yang Xia, Yujuan Niu, Xinguang Liu, Xuemei Qin

**Affiliations:** 1 Department of Hematology, Second Clinical Medical College of Gansu University of Chinese Medicine, Lanzhou First People’s Hospital, Lanzhou, China; 2 Department of Hematology, Qilu Hospital, Cheeloo College of Medicine, Shandong University, Jinan, China

**Keywords:** anti-myeloma therapy, autologous hematopoietic stem cell transplantation, electrolyte replacement, Fanconi syndrome, multiple myeloma

## Abstract

**Background:**

Fanconi syndrome (FS) is a rare renal complication of monoclonal gammopathies including multiple myeloma (MM), typically diagnosed before or at the time of MM presentation. Onset and progression of FS during anti-myeloma treatment are extremely rare and easily misdiagnosed, resulting in delayed electrolyte correction and clinical deterioration.

**Case presentation:**

We report a 55-year-old woman with κ light chain MM who developed FS during induction therapy and experienced recurrence after autologous stem cell transplantation (ASCT). Initial workup showed subclinical tubular dysfunction with renal glycosuria and elevated tubular markers, which was initially overlooked. Shortly after induction therapy, she developed severe and recurrent hypokalemia and hypophosphatemia accompanied by progressive gastrointestinal symptoms despite hematological remission. A multidisciplinary consultation confirmed MM-associated FS. Electrolyte replacement, particularly phosphate supplementation, rapidly relieved her symptoms. With ongoing anti-myeloma therapy, glycosuria gradually resolved. Following ASCT, FS recurred but resolved with supportive care. The patient has remained in remission during continued follow-up.

**Conclusion:**

Anti-myeloma therapy may transiently induce or exacerbate FS, especially in patients with pre-existing renal tubular dysfunction, likely due to synergistic tubular toxicity of anti-myeloma agents. Early recognition, frequent electrolyte monitoring and prompt correction, as well as multidisciplinary collaboration, are crucial for optimal management of FS.

## 1 Introduction

Multiple myeloma (MM) is a hematologic malignancy characterized by clonal proliferation of plasma cells. Renal impairment is frequent in MM, with 20%–40% of newly diagnosed patients demonstrating varying degrees of renal dysfunction (1). Common renal pathologies include light−chain cast nephropathy, amyloidosis, and monoclonal immunoglobulin deposition disease (2). Light−chain proximal tubulopathy (LCPT) is rare and typically presents as Fanconi syndrome (FS).

FS is a clinical disorder caused by impaired proximal tubular transport, leading to defective reabsorption of multiple solutes. Key features include normoglycemic glycosuria, hyperphosphaturia, hypophosphatemia, aminoaciduria, hypouricemia, hypokalemia, and proximal renal tubular acidosis (RTA). Etiologies are hereditary or acquired; monoclonal gammopathy is a leading cause of adult−onset acquired FS, termed light chain−associated FS (LCFS). Most LCFS cases occur with monoclonal gammopathy of undetermined significance (MGUS) or MM, with rare associations with Waldenström macroglobulinemia (WM), lymphoplasmacytic lymphoma (LPL), and chronic lymphocytic leukemia (CLL) (3). FS is usually diagnosed before or at MM diagnosis (4); onset during anti−myeloma therapy is rare, and non−specific gastrointestinal symptoms are often misdiagnosed as drug−related adverse events. Here, we report a rare case of MM-associated FS that developed during induction therapy and recurred after autologous stem cell transplantation (ASCT), and a relevant literature review is also conducted herein to improve the clinical recognition and management of this disorder.

## 2 Case report

On September 20, 2024, a 55−year−old woman was admitted to Qilu Hospital, Shandong University, China, with a 5−month history of lower back and rib pain. She denied fatigue, nausea, or anorexia, and had no history of hypertension, diabetes mellitus, or chronic kidney disease. Physical examination was unremarkable. Laboratory results are summarized in **Table 1**.

**Table 1:** Laboratory test data before and after chemotherapy and ASCT at diagnosis of FS.

Laboratory parameter (reference range)	Before chemotherapy	After chemotherapy	Before ASCT	After ASCT
Fasting plasma glucose Glucos (3.9-6.1 mmol/L)	4.65	4.12	6.0	5.32
Serum sodium (137-147 mmol/L)	143	142	144	139
Serum Potassium (3.5-5.3 mmol/L)	3.83	3.16	3.69	2.94
Serum Chloride (99-110 mmol/L)	108	112	108	112
Serum Calcium (2.11-2.52 mmol/L)	2.54	2.02	2.1	2.21
Serum Phosphate (0.65-1.1 mmol/L)	1.36	0.45	0.98	0.36
Creatinine (53-97 umol/L)	103	70	54	42
Glomerular filtration rate (ml/min)	53.22	84	102	111
Serum uric acid (155-357 umol/L)	100	67	82	72
β_2_ microglobulin (0.7-1.8mg/L)	6.42	1.58	2.45	NA
serum immunofixation electrophoresis	κ+	–	–	NA
Serum κ free light chain (3.3-19.4ng/L)	20071.99	181.45	29.65	NA
Arterial blood gas analysis				
PH (7.35-7.45)	7.38	7.36	NA	7.4
pSO_2_ (35-45mmHg)	33	26	NA	32
PO_2_ (80-100mmHg)	101	122	NA	63
sO_2_(95-98%)	98.8	100	NA	92.1
BE (-3-3mmol/L)	-4.8	-9.5	NA	-4.4
HCO_3_^-^ (21-28mmol/L)	19.5	14.7	NA	19.8
Anion gap(8-16mmol/L)	8.5	11.3	NA	7.2
Urinalysis				
pH (5.4–8.4)	6.0	5.5	5.0	5.5
Urine Protein	2+	1+	–	1+
Urine sugar	1+	4+	1+	3+
Total 24-hour urinary protein (g /24h)	N/A	2.04	1.02	NA
24-hour urine electrolytes				
Potassium (50–100 mmol/L)	NA	33.48	NA	NA
Sodium (130–217 mmol/L)	NA	220	NA	NA
Chloride(173–250 mmol/L)	NA	123	NA	NA
Phosphorus (3.5–8.4 mmol/L)	NA	10.75	NA	NA
Glucose(mmol/L)	NA	202.68	NA	NA
Creatinine (7000-14000 µmol/L)	NA	1396	NA	NA
Fractional excretion of phosphate (FEP) 10%–20%)	NA	49%	NA	NA
Renal tubular function				
Urinary microalbumin(<30 mg/L)	129	21.8	NA	NA
Urinary α1-microglobulin(<20 mg/L)	194	86.5	NA	NA
Urinary β2-microglobulin(<0.22 mg/L)	144	64	NA	NA

NA, not applicable.

At admission, complete blood count and liver function tests were normal. Serum creatinine level was 103 μmol/L (reference range, 53-97 μmol/L), serum uric acid level was 100 μmol/L (reference range,155-357 μmol/L), estimated glomerular filtration rate(eGFR)was 53.22 mL/min and serum calcium level was 2.54 mmol/L (reference range, 2.11-2.5 mmol/L). Serum sodium, chloride, potassium, phosphorus, fasting glucose, and lactate dehydrogenase were within normal limits. β2-microglobulin level was 6.42 mg/L (reference range, 0.7-1.8mg/L). Urinalysis showed pH 6.0, glucose 1+, occult blood 2+, and protein 2 +. Arterial blood gas analysis indicated compensated metabolic acidosis: pH 7.38, PaCO_2_ 33 mmHg, PaO_2_ 101 mmHg, anion gap 8.5 mmol/L, actual base excess -4.8 mmol/L, and actual bicarbonate level was 19.5 mmol/L. Renal tubular function tests revealed urinary microalbumin level was 129 mg/L (reference range, <30 mg/L), α1-microglobulin level of 194 mg/L (reference range, <20 mg/L), and β2-microglobulin level was 144 mg/L (reference range, <0.22 mg/L). Serum immunofixation electrophoresis identified a κ light chain type. The serum free λ light chain level was 4.8 ng/L (reference range, 5.71-25.3 ng/L), the serum free κ light chain level was 20071.99 ng/L (reference range, 3.3-19.4 ng/L), and the κ/λ ratio was 4181.6646 (reference range, 0.26-1.65).

Bone marrow examination showed 32% immature plasma cells, and immunophenotyping revealed 19.5% abnormal plasma cells expressing CD38, CD138, CD56dim, CD27dim, BCMA, and cytoplasmic κ light chains. MM-FISH analysis was negative. Whole-body ECT and spinal CT demonstrated osteoporosis with fractures of the T9 vertebra and bilateral ribs. Bone mineral density showed a T-score was -3.1.

Based on these findings, the patient was diagnosed with κ light chain multiple myeloma, ISS stage III. Renal glycosuria and elevated tubular markers suggested subclinical tubular dysfunction, which was initially overlooked. After written informed consent, she started VRD therapy (bortezomib, lenalidomide, dexamethasone) and received denosumab 120 mg for bone disease. On day 5 of treatment, serum potassium level of 3.16 mmol/L, serum phosphorus level of 0.45 mmol/L, serum creatinine decreased to 70 μmol/L, and eGFR increased to 84 mL/min. The patient reported no significant discomfort and was discharged after potassium supplementation alone.

After one cycle, bone pain resolved, serum free κ light chain fell to 181.45 ng/L, and renal function normalized. However, she developed nausea, vomiting, anorexia, and limb numbness. Biochemical testing showed hypokalemia (3.11 mmol/L) and hypophosphatemia (0.34 mmol/L). Symptoms were initially attributed to drug toxicity and insufficient intake; antiemetics and electrolyte replacement were given. Electromyography confirmed bortezomib−induced peripheral neuropathy. Treatment was switched to IRD (ixazomib, lenalidomide, dexamethasone) in cycle 2, but symptoms persisted. Preparing for cycle 3, she presented with poor performance status and severe malnutrition. She reported a progressive worsening of nausea, vomiting and anorexia, which led to a marked reduction in oral intake, severely disrupted her daily activities and resulted in a significant decline in her self-assessed quality of life. Repeat testing showed severe electrolyte disturbances: potassium 3.09 mmol/L, phosphorus 0.24 mmol/L (calcium and magnesium normal). Arterial blood gas: pH 7.36, base excess −9.5 mmol/L, bicarbonate 14.7 mmol/L. Urinalysis: pH 5.5, glucose 4+, occult blood 3+, protein 1 +.

For a patient with persistent unexplained electrolyte disturbances and progressive gastrointestinal symptoms despite hematologic remission of MM, we organized a multidisciplinary consultation involving the Departments of Hematology, Nephrology, and Endocrinology. A diagnosis of MM-related FS was established based on glycosuria, hypophosphatemia, hypokalemia, metabolic acidosis, and hypouricemia. 24-hour urine biochemistry panel showed potassium 33.48 mmol/L (reference range, 50–100 mmol/L), sodium 220 mmol/L (reference range, 130–217 mmol/L), chloride 123 mmol/L (reference range, 173–250 mmol/L), phosphorus 10.75 mmol/L (reference range, 3.5–8.4 mmol/L), glucose 202.68 mmol/L, creatinine 1396 µmol/L (reference range,7000-14000 µmol/L), and fractional excretion of phosphate (FEPO_4_) is 49% (reference range, 10%–20%). Increased FEPO_4_ further supported the diagnosis of FS. Urinary uric acid, bicarbonate, and aminoaciduria were not available. Subsequently, she received oral and intravenous potassium and phosphorus supplementation, symptoms improved within 3 days. She continued regular IRD therapy plus oral electrolyte replacement for 4 months; electrolytes normalized, and glycosuria/proteinuria resolved. The patient reported recovery of the ability to perform basic daily activities and a marked improvement in overall health status.

**Figure 1:**
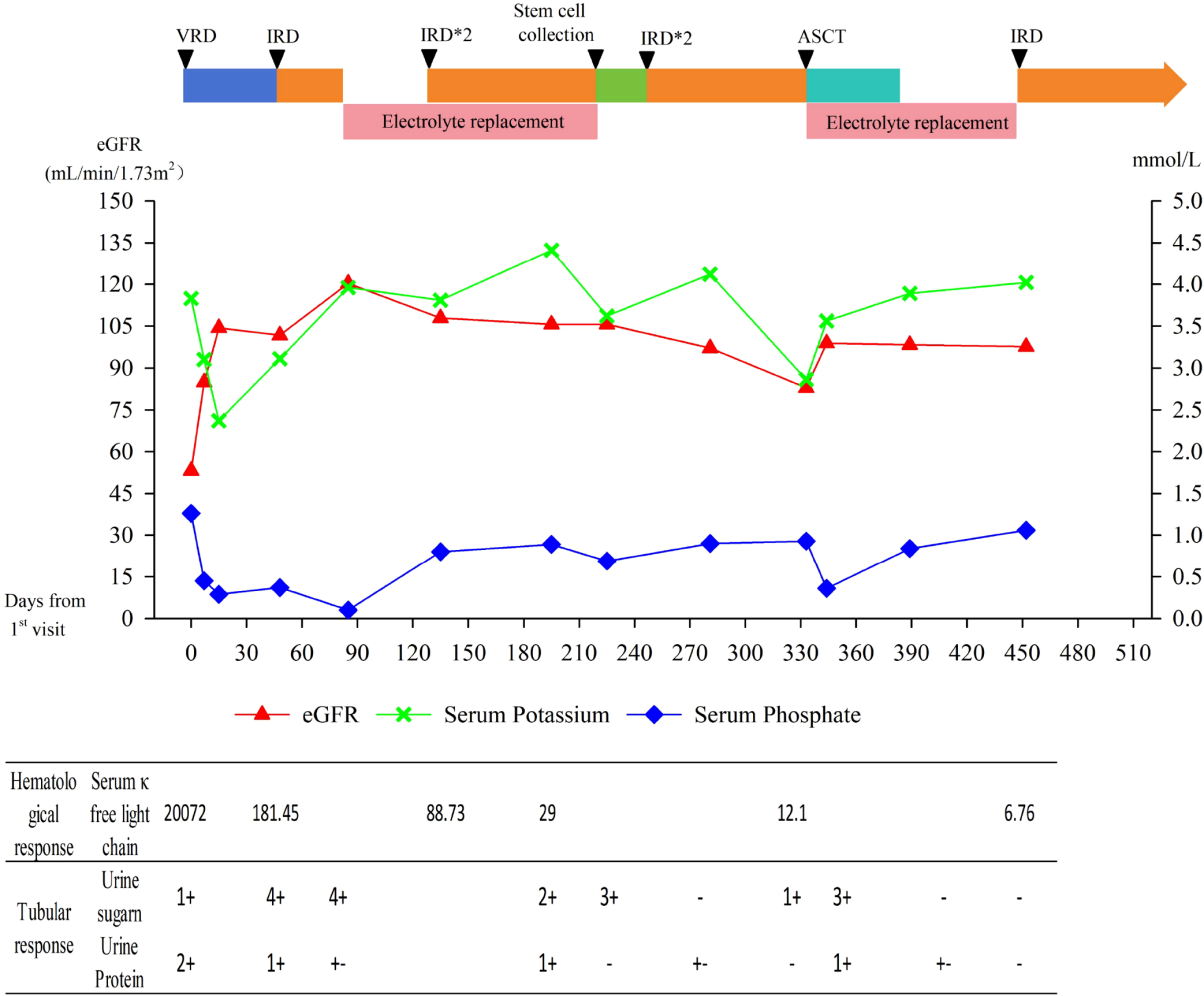
Clinical course of the patient. VRD, bortezomib, lenalidomide, dexamethasone; IRD, ixazomib, lenalidomide, dexamethasone; ASCT, autologous stem cell transplantation; eGFR, estimated glomerular filtration rate.

After 6 cycles, she achieved VGPR, but urinalysis showed recurrent glycosuria. On August 29, 2025, she underwent ASCT with melphalan conditioning. On post - ASCT day 5, hypokalemia, hypophosphatemia, nausea, vomiting, and anorexia recurred (urine protein 1+, glucose 3+), indicating FS recurrence. Symptoms resolved with electrolyte replacement. She remains stable in follow−up. The patient’s clinical course is illustrated in **Figure 1**.

## 3 Literature review

A systematic review of LCFS was performed via PubMed, Embase, and Cochrane Library (inception to December 2025) using keywords: “Fanconi syndrome”, “light chain proximal tubulopathy”, “multiple myeloma”, “monoclonal gammopathy”.

The inclusion criteria were case reports and case series that meet the diagnosis of both monoclonal gammopathy and FS, with relatively complete records of treatment and prognosis. Exclusion criteria were the presence of another cause of FS, especially the use of any drugs known to induce proximal tubular (PT) dysfunction. A total of 18 case reports and 6 case series were included (5–26) (summarized in **Tables 2**, **3**). The limitations of this review lie in its nature as a narrative synthesis and the potential publication bias inherent in case reports.

**Table 2:** Cases report of LCFS.

Reference	Age (years)	Sex	Underlying disease	Type of monoclonal gammopathy	Time to presentation	Typical symptoms	Sequence of FS and MG onset	Treatments	Hematological response	Renal response	Follow-up
(5)	38	Male	MM	NA	3 years	Fatigue, nocturia	Simultaneous	Supplement treatment cortisone	NA	Progression	1year
(6)	40	Male	MCUS	κ	1 year	Fatigue, thirst, frequency of micturition, paraesthesiae, myasthenia	Simultaneous	Cyclophosphamide Supplement treatment	NA	Stable	3years
(6)	42	Male	MCUS	κ	4 years	Fatigue, muscular pains	Simultaneous	Supplement treatment	NA	Stable	10 months
(7)	39	Female	MM	κ	2 years	Glycosuria, low back pain	FS before MG	MP, vincristine	VGPR	Improved	10 months
(8)	66	Male	MM	κ	NA	Rib pain, nausea, anorexia	Simultaneous	MP	VGPR	Improved	4 years
(9)	78	Male	MM	IgG-k	2 months	Proteinuria, renal insufficiency	FS before MG	MP	PR	Stable	3 years
(10)	80	Male	MM	IgG-k	NA	Bone pain, myasthenia	Simultaneous	chemotherapy	NA	Stable	NA
(11)	58	Male	MM	IGD-λ	3 months	Acute renal failure	Simultaneous	VD	PR	NA	30 months
(12)	76	Male	MCUS	IgA-κ	1 year	Renal failure	Simultaneous	High-dose dexamethasone→ VD	VGPR	Renal function improved, renal tubular function Stable.	18 months
(12)	54	Male	MCUS	κ	NA	Renal insufficiency, proteinuria	Simultaneous	High-dose dexamethasone→VD	PR	Renal function improved, renal tubular function Stable.	18 months
(13)	76	Male	WM	IgM-κ	NA	Bone pain, lower limb edema	Simultaneous	Rituximab+VD	PR	Renal tubular function Stable.	NA
(14)	68	Male	MM	λ	NA	Proteinuria, acute renal failure	Simultaneous	VTD	CR	Improved	1 year
(15)	48	Female	MM	κ	6 months	Proteinuria	Simultaneous	CD→ASCT	VGPR	Renal tubular function Stable.	1 year
(16)	75	Male	MM	κ	NA	Bone pain, myasthenia	Simultaneous	VD	PR	Renal tubular function Stable.	5 months
(17)	64	Female	SMM	κ	10 years	Back pain, myasthenia	Simultaneous	Prednisolone	NA	Stable.	NA
(18)	45	Female	MM	κ	1 month	Fatigue, anorexia	Simultaneous	VTD	CR	Renal function improved	NA
(19)	64	Male	MCUS	κ	3 years	Renal insufficiency, hypokalemia	Simultaneous	VCD→ASCT	CR	Improved	9 months
Our case	55	MM	MM	κ	5 months	Nausea, vomiting, anorexia	FS after MG	VRD→IRD→ASCT	CR	Improved	15 months

MM, multiple myeloma; SMM,smoldering multiple myeloma; MCUS,monoclonal gammopathy of undetermined significance; WM,Waldenstrom macroglobulinemia; MG,monoclonal gammopathy; FS, Fanconi syndrome; CR, complete response; VGPR, very good partial response; PR, partial response; ASCT, autologous stem cell transplantation; MP, melphalan, prednisone; VD, bortezomib, dexamethasone; VTD, bortezomib, thalidomide, dexamethasone; CTD, cyclophosphamide, thalidomide, dexamethasone; VTD,bortezomib, thalidomide, dexamethasone; VCD, bortezomib, thalidomide, dexamethasone; VRD, bortezomib, lenalidomide, dexamethasone; IRD, ixazomib, lenalidomide, dexamethasone; NA, not applicable.

**Table 3:** Case series of LCFS.

Reference	Case(n)	Age (years)	Sex	Underlying disease	Type of monoclonal gammopathy	Typical symptoms	Time to presentation (months)	Sequence of FS and MG onset	Treatments	Hematological response	Renal outcomes
(21)	11 (Full-blown FS 9/11)	55(36-74)	Male 5 Female 6	MCUS 3 MM 5 MCUS/MM 3	IgG-κ,κ 2 IgA-κ 2 K 7	Renal failure7/11 Bone pain 2/11	74.5(5-144)	FS before MG 2 Simultaneous 9 FS after MG 0	Alkeran +prednisone 2 VAD→VMCP+ BVAP 1 Other chemotherapy 1 a-Interferon 2 Without any treatment 3 Supplement treatment only 2	NA	NA
(22)	32	58 (31-81)	Male 22 Female 10	MM 10 WM 2 SMM 6 MCUS 14	IgA-κ 3 IgG-κ 9 IgM-κ 2 K 15 IgG-λ 3	Bone pain 15/32(47%) Fatigue 7/32(22%) Renal Insufficiency, Proteinuria, or Glycosuria 10/32(31%)	NA	NA	Chemotherapy 22/32	NA	ESRD 5
(23)	8 (complete FS 7/8)	45.5 (23-68)	Male 3 Female 5	MM 2 MCUS 6	IgG-κ 2 K 5 IgA-λ 1	Bone pain6/8 Nocturia, Thirst 2/8 Osteomalacia 4/8 Stress fracture4/8	39(2 - 96)	Simultaneous 8/8	MP×1→ VD×6 1 MT×1 1 Supplement treatment only 6 Abandon treatment 1	PR 1/1	Renal function progression 8/8 ESRD 0/8
(24)	49 (complete FS 17/49)	58(37-83)	Male 30 Female 19	MGUS 13 SMM 25 MM 7 WM 4	IgG-κ 21 IgM- κ 4 IgA-κ 5 K 16 IgG -λ 2 IgA-λ 1	Renal Insufficiency 65%	12	NA	ASCT 14 Bortezomib based 11 MiDs 7 Alkylating 6	CR 4/38 VGPR 10/38 PR 20/38 SD 4/38	Renal response 34/38 Tubular response 12/38 ESRD 9/38
(25)	22 (complete FS 17/22)	49 (30–76)	Male 8 Female 14	MM 6 sMM 2 MCUS 13 WM 1	Light chain isotype κ20 λ 2 Heavy chain isotype IgG 6 IgA 4 IgM 1 Light chain only 11	Bone pain 72.7% Hypodynamia 40.9% Myasthenia 22.7%, Proteinuria 36.4% Polyuria 6%	35 (4–85)	NA	Bortezomib based 7 IMiDs 9 ASCT 2	CR 3/18 VGPR 2/18 PR 6/18 SD3/18	Renal response 4/11 Tubular response10/15 ESKD 0
(26)	26 (Half combined complete FS)	54.7 (40–69)	Male 11 Female 15	MGRS 14 MM 10 WM 1 PPCL 1	Light chain isotype κ22 λ 4 Heavy chain isotype IgG 7 IgA 2 IgM 1 Light chain only 16	Fatigue 22/23 (95.7%) Ostealgia 23 (88.5%) Nocturia 11/18 (61.1%) Stress fracture 6 (23.1%)	NA	NA	Bortezomib based 6 IMiDs 10 Melphalan based 2 ASCT 1	CR 3/12 VGPR/PR 6/12 SD/PD 3/12	Renal response7/12 Tubular response 8/12 ESKD 0/12

MM, multiple myeloma; SMM, smoldering multiple myeloma; MCUS, monoclonal gammopathy of undetermined significance; WM, Waldenstrom macroglobulinemia; MG, monoclonal gammopathy; FS, Fanconi syndrome; CR, complete response; VGPR, very good partial response; PR, partial response; ASCT, autologous stem cell transplantation; BVAP, BCNU, VM16, adriblastine, prednisolone; VAD,vincristine, adriblastine, dexamethasone;VMCP, VM16 (in the place of vincristine), melphalan, cyclophosphamide, prednisolone; MP, melphalan plus prednisone; VD, bortezomib, dexamethasone; MT, melphalan, thalidomide; NA, not available.

LCFS is a recognized but rare renal complication of monoclonal gammopathies such as MM. Its pathogenesis is associated with the deposition of abnormal free light chains (FLCs) produced by monoclonal plasma cells in proximal tubular cells of the kidney. Under normal physiological conditions, small amounts of FLCs are reabsorbed via the megalin/cubilin scavenger receptor on the apical surface of proximal tubular epithelium and subsequently degraded through the lysosomal pathway. In the setting of dysproteinemias, however, the proximal tubules endocytose excessive FLCs, predominantly κ-type light chains, whose variable domains possess intrinsic physicochemical properties that confer resistance to proteolysis and promote self-aggregation and crystal formation. The reabsorbed but undegraded monoclonal light chains accumulate in the proximal tubules in either crystalline or non-crystalline forms, a condition termed light chain proximal tubulopathy (LCPT) (27). Approximately 68% of LCPT cases manifest as FS (28).

FS is classically characterized by normoglycemic glycosuria, aminoaciduria, hypophosphatemia or hyperphosphaturia, hypouricemia, and RTA. When all these features are present, the condition is classified as full-blown FS; if one or two features are missing as well as specific PT injuries confirmed by kidney biopsy, incomplete FS was diagnosed (24). Full-blown FS is typically more common than incomplete FS (21). Most patients also exhibiting proteinuria and varying degrees of renal dysfunction at initial diagnosis (24). Proteinuria is considered a feature of FS, likely resulting from impaired proximal tubular reabsorption. The diagnosis of LCFS requires the presence of classic FS-related proximal tubular dysfunction, confirmation of monoclonal light chains by serum/urine free light chain assays or immunofixation electrophoresis, exclusion of other secondary FS etiologies, and is further supported by pathological findings of light chain-induced proximal tubular injury on renal biopsy.

In most cases, FS is an early manifestation of monoclonal gammopathy (4). FS is usually diagnosed before or concurrently with monoclonal gammopathy, with a clinical history ranging from several months to years before diagnosis. The most common manifestations include renal insufficiency, proteinuria, glycosuria, fatigue, bone pain, and osteomalacia (5–26) (**Tables 2**, **3**). Delayed diagnosis is common until typical features emerge. Late-onset FS is often drug-related, such as cisplatin and ifosfamide, which cause direct damage to proximal tubular epithelial cells via metabolites, leading to generalized solute reabsorption dysfunction (29). In recent years, several cases of lenalidomide-induced FS have been reported, typically occurring months after treatment initiation, with a clear temporal association with lenalidomide exposure but no correlation with FLC levels. Pathological findings show pan-tubulopathy, although the precise mechanism remains unclear. Notably, this adverse effect is usually reversible upon drug discontinuation (30, 31).

Over the past few decades, management of LCFS has mainly consisted of electrolyte replacement and correction of acidosis to relieve symptoms. Conventional chemotherapy based on alkylating agents shows low efficacy and carries a risk of secondary malignancies (22). In recent years, bortezomib-based regimens have been shown to stabilize or improve renal and tubular function and are currently recommended as first-line therapy for LCFS (25). Renal response occurs only in patients achieving at least a partial hematologic response (PR), and early diagnosis and treatment significantly shorten the time to tubular function recovery (24).

Regarding the role of ASCT in LCFS, some studies suggest that chemotherapy plus ASCT yields superior renal or tubular responses compared with chemotherapy alone (19). Nevertheless, MM remains incurable, and FS may recur with disease relapse, requiring further investigation of optimal therapeutic strategies (32).

## 4 Discussion

In this case, the patient was diagnosed with MM. At initial presentation, she exhibited typical features of proximal renal tubular dysfunction, including renal glycosuria and proteinuria. Urinary microalbumin mainly reflects early glomerular filtration barrier impairment but also indicates defective proximal tubular reabsorption of small-molecular-weight proteins. Urinary α_1_-microglobulin is a specific marker of proximal tubular injury, and its elevation directly indicates impaired reabsorptive capacity. Urinary β_2_-microglobulin is a highly sensitive indicator of proximal tubular dysfunction, as even mild injury leads to a marked increase due to impaired reabsorption and degradation. In this patient, all three key biomarkers of proximal tubular injury were significantly elevated, further supporting the diagnosis of proximal tubular damage. After excluding secondary etiologies including hereditary disorders, drug toxicity, and autoimmune diseases, LCPT was suspected clinically.

Shortly after anti-myeloma therapy, the patient rapidly developed hypokalemia and hypophosphatemia. Severe malnutrition and rapid calorie repletion were absent before treatment, excluding refeeding syndrome. Persistent diarrhea or vomiting was not present during electrolyte disturbances, excluding gastrointestinal losses. Drug-induced diarrhea was excluded because no diarrhea occurred after starting anti-myeloma therapy. In addition, renal function gradually improved without hyperkalemia or hyperphosphatemia, making tumor lysis syndrome (TLS) unlikely.

Therefore, combined with features including hypophosphatemia, hypokalemia, increased urinary phosphate excretion, proximal renal tubular acidosis, and hypouricemia, the patient fulfilled the diagnostic criteria for FS. Although the absence of important parameters such as aminoaciduria and urinary uric acid may limit the comprehensive evaluation of tubular function, it does not negate the diagnosis.

Renal biopsy is the gold standard for diagnosing LCFS, as it identifies characteristic monoclonal light chain deposition in proximal tubules, confirms the diagnosis, and differentiates LCFS from other causes of FS. Its diagnostic value is well documented in the literature (27). Unfortunately, renal biopsy was not performed because the patient’s renal function was relatively preserved at the time and clinical awareness of the disease was limited. Despite the lack of pathological confirmation, the patient’s clinical manifestations were consistent with LCFS in the setting of pre−existing LCPT. This case underscores the necessity for more comprehensive evaluation of such patients in the future.

The development of FS during induction therapy in this patient is a rare clinical event. We analyzed its underlying pathogenesis in detail. First, pre-existing proximal renal tubular dysfunction served as a predisposing factor. Monoclonal light chains had already induced subclinical and compensated tubular injury, providing the pathological basis for subsequent FS. Second, the synergistic tubular toxicity of anti-myeloma agents likely contributed. Lenalidomide and bortezomib are core components of anti-myeloma treatment. Lenalidomide has been previously associated with FS (30, 31) and may play a central role in triggering proximal tubular damage. In animal studies, bortezomib impairs proteasome function and cellular metabolism in tubular epithelial cells, thereby exerting direct tubular toxicity (33). The combination of these two agents may have aggravated tubular injury, resulting in defective reabsorption of multiple solutes and the typical clinical features of FS. Third, disordered calcium-phosphate homeostasis further compromised tubular ion transport. Denosumab, a RANKL inhibitor, markedly reduces osteoclast activity and can cause severe hypocalcemia and hypophosphatemia (34). Phosphate regulates tubular epithelial function via Na+-Pi cotransporters and calcium channels and abnormal calcium-phosphate levels directly impair tubular reabsorption (35). Denosumab likely precipitated calcium-phosphate imbalance and worsened tubular ion handling. Notably, FS progressed despite hematological remission, partly because electrolyte disturbances were not corrected promptly. In our patient, early hypophosphatemia was not adequately treated with phosphate supplementation. Sustained hypophosphatemia caused intestinal smooth muscle dysfunction, leading to nausea, anorexia, and reduced oral intake, while further injuring proximal tubules and worsening reabsorptive capacity. This created a vicious cycle: renal electrolyte loss → hypokalemia/hypophosphatemia → gastrointestinal symptoms/tubular injury → reduced oral intake/renal electrolyte loss → severe electrolyte disturbance. Subsequent electrolyte replacement, especially phosphate supplementation, markedly alleviated symptoms, supporting this mechanism.

However, the complexity of this case did not end with the induction phase. A particularly challenging observation was that the patient experienced a recurrence of FS only a few days after high−dose melphalan conditioning. Before ASCT, renal glycosuria reappeared in our patient, despite complete hematologic remission. This indicates that although effective treatment suppressed light chain production, tubular recovery was incomplete and residual tubular defects persisted in some patients (24). Melphalan is an alkylating agent secreted and reabsorbed via renal tubules and is generally considered to have low nephrotoxicity. Nevertheless, case reports of melphalan−induced acute kidney injury exist, especially in patients with pre−existing tubular dysfunction (29). The development of FS in this patient likely occurred through a similar mechanism. Janina et al. described 5 cases of acute FS following ASCT, with an average onset of 10.2 days. While one case could not exclude lenalidomide involvement, the pathogenesis remained unclear in others. All cases recovered rapidly after electrolyte replacement, leading the authors to hypothesize that this phenomenon may represent a self-limiting and transient feature after ASCT. However, based on the reported laboratory data, all five patients had varying degrees of renal dysfunction (eGFR 41–105 mL/min) before ASCT, and four had glucosuria, suggesting that these cases may be similar to ours, with pre-existing tubular dysfunction present before treatment, although this was not recognized by the authors. Notably, only one patient was diagnosed with LCPT by renal biopsy prior to ASCT [36].

In summary, we report a rare case of MM-related FS. The patient developed and progressed with FS during anti-myeloma induction therapy on the basis of pre-existing subclinical renal tubular dysfunction, with recurrence following ASCT. Through review of the entire clinical course and analysis of relevant literature, we have further strengthened the holistic management of FS.

This study has several limitations: first, it is a single case report with a limited sample size, which restricts the generalizability of the conclusions; second, renal biopsy was not performed, so histological evidence of tubular injury is lacking, and incomplete key quantitative data, such as the fractional excretion of phosphate, uric acid, bicarbonate, aminoaciduria, and urea nitrogen, limited the comprehensive evaluation of renal tubular damage; third, no *in vitro* or *in vivo* experimental data were available to verify the proposed hypothesis of synergistic tubular toxicity. The generalizability and causal relationship require validation in prospective studies.

## 5 Conclusion

LCFS is a rare renal complication of multiple myeloma. Renal glycosuria may be its sole presentation. When glycosuria occurs in the setting of normal blood glucose, comprehensive evaluation of renal tubular function should be performed to investigate the possibility of FS. Early identification of FS and timely electrolyte replacement are critical. For patients with pre-existing renal tubular injury or confirmed FS, individualized adjustment of anti-myeloma regimens is necessary to avoid synergistic renal tubular toxicity induced by multiple medications. It should also be highlighted that FS may persist as a chronic complication even after the effective remission of the primary disease, hence long-term renal function follow-up is recommended to monitor recurrent renal tubular damage.

## Data Availability

The original contributions presented in the study are included in the article/supplementary material. Further inquiries can be directed to the corresponding author.
